# Design and Durability Assessment of Restoring Mortar for Concrete Heritage

**DOI:** 10.3390/ma14164508

**Published:** 2021-08-11

**Authors:** Judite Miranda, Hugo Costa, Jónatas Valença, Ricardo do Carmo, Eduardo Júlio

**Affiliations:** 1 CERIS—Civil Engineering Research and Innovation for Sustainability, 1049-001 Lisbon, Portugal; judite.botas@tecnico.ulisboa.pt (J.M.); hcosta@isec.pt (H.C.); carmo@isec.pt (R.d.C.); eduardo.julio@tecnico.ulisboa.pt (E.J.); 2 Instituto Superior Técnico, Universidade de Lisboa, 1049-001 Lisbon, Portugal; 3 ISEC-Polytechnic Institute of Coimbra, 3045-093 Coimbra, Portugal; 4 Associação do Instituto Superior Técnico para a Investigação e Desenvolvimento, Universidade de Lisboa, 1049-001 Lisbon, Portugal

**Keywords:** concrete heritage, restoration mortar, colour, texture, performance, durability, service life

## Abstract

Interventions in concrete heritage deal with challenges related to conservation, and must be performed from an integrated restoration perspective. In addition to the material technical performance, the aesthetic compatibility between the repair and the structure, in terms of colour and texture, needs to be ensured. Therefore, the characterisation of the restoration mortar concerning colour match and aging, and the mechanical and durability performances, is essential. In this article, the long-term behaviour of restoration mortar, previously designed and produced by the addition of pigments to white and grey cement-based reference mortar, is evaluated. The durability properties, colour change due to aging, and service life are estimated and analysed. An experimental program is performed to characterise the following properties: (i) water capillary absorption; (ii) accelerated carbonation; (iii) migration of chloride ions; (iv) electrical resistivity; and (v) shrinkage. The colour evolution, when exposed to carbonation, is measured through image processing. The obtained results allow the establishment of a correlation between durability and design parameters. Finally, service life considering deterioration due to steel corrosion is estimated, considering the carbonation resistance and the chloride diffusion values. It is concluded that the W/C ratio influences not only most of the characterised parameters, but also the type and content of the pigment. Furthermore, no colour variation due to carbonation is detected.

## 1. Introduction

The preservation of “concrete heritage” is an increasing issue in society. The design and performance of restoration mortar still attracts limited interest from the scientific community. Interventions in exposed concrete constructions often lead to unsatisfactory results from a restoration point of view, with them not complying with aesthetic criteria. These criteria demand the production of mortar of a planned colour through pigmentation, addition, and specific application procedures, to positively address the singularities of a restoration project. Intervention allows the preservation of historical and cultural significant construction and of the material itself. The intended aesthetic compatibility is difficult to achieve, and often solved empirical by the knowledge of the technicians starting from commercial solutions [1,2,3]. Furthermore, cracks due to bond mismatch and shrinkage are also frequently identified. The commercially available solutions for structural rehabilitation of concrete are generally cement-based mortar, with selected sand and specific additions, ready to be mixed with water and then applied. The additions and admixtures are generally intended to reduce shrinkage, to improve workability and thixotropy, to reduce sulphate attack, and to increase mechanical and adhesive strengths, usually through the addition of fibres. Despite some important properties assured by the specifications of these solutions, their application commonly results in huge incompatibility regarding colour, texture, elastic behaviour, and durability requirements. Furthermore, the need for aesthetic finishing in concrete heritage requires several techniques that are not compatible with the fibres of the mortar. Therefore, the lack of suitable commercial material justifies the need to find adequate alternative solutions. New cementitious restoration material can be developed and proposed, which should be specially formulated to meet all mechanical, time-dependent, durability, and aesthetic requirements. These must be based on scientific and technical solutions [4,5].

The degradation of concrete is commonly caused by a combination of external and internal factors. It results from a complex process mainly related to the physicochemical properties of concrete and to the exposed conditions [6]. These degradation processes or reactions modify a material’s ability to perform its functions as initially intended. Concerns about the durability and the long-term performance of restoration mortar are an important issue for many applications, and can be the reason to limit the use of some additions. Thus, it becomes essential to characterise the performance of restoration mortar in terms of water absorption, carbonation depth, chloride migration, and shrinkage.

The service life of a concrete structure is defined as the period during which it meets the requirements for safety, functionality, and aesthetics without requiring significant maintenance costs [7]. The service life for each structure must be defined, usually as 50 or 100 years, and must be considered during the design stage. One of the major factors that affects the service life of reinforced concrete structures is performance against the corrosion of reinforcement steel. To accomplish these requirements, is it necessary to know the mechanical, time-dependent, and durability properties of the concrete. The durability of concrete is frequently related to its ability to resist penetration or diffusion of substances in the environment. This ability depends on the internal structure of the concrete, mainly on the binder matrix and on transport properties. Normal aggregates have a minor influence on this performance, because they are usually inert and have reduced open porosity. However, abovementioned commercial cement-based solutions for concrete restoration are commonly designed for strength purposes and, as well as providing no adequate chromatic and aesthetic compatibility for concrete surfaces, the durability requirements are not properly specified. In addition, no research studies exist in the literature regarding design methods and durability performance for repairing mortar for structural purposes. Therefore, the present study examines an ongoing project that aims to develop innovative methods and solutions that combine strength, aesthetics, and durability requirements.

The main objective of this article is the evaluation of long-term behaviour of restoration mortar, specifically designed for interventions in concrete heritage. The mortar was previously developed and produced through the addition of different proportions of pigments (yellow, red, black ferrous oxides, and blue cobalt oxide) to reference repairing mortar, based on white and grey cement. The characteristics of durability, colour change due to aging, and estimated service life are analysed, depending on the type and content of adopted pigment. The influence of water-to-cement ratio was also considered. An experimental program was planned to properly characterise the mechanical strength, shrinkage, and main durability properties. The definition and quantification of the behaviour regarding colour change of restoration mortar subjected to accelerated carbonation is also analysed. The analysis aims to establish correlations and to evaluate the influence of the type of added pigment on durability parameters. Finally, service life related to the deterioration caused by the reinforcement corrosion is estimated, as well as the minimum cover required to provide a proper protection for XC and XS exposure classes, as defined in EN 206 [8].

## 2. Materials and Methods

The experimental study was developed for: (i) reference white and grey cement-based mortar with the different W/C ratios; (ii) grey restoration mortar produced by the addition of black pigment to the reference white and grey mortar and; (iii) coloured restoration mortar, produced by the addition of three pigments—yellow, red, and blue—with different chemical bases to the reference white mortar. Shrinkage and durability properties were characterised. The yellow, red, and black inorganic pigments are based on iron oxide, FeHO_2_, Fe_2_O_3_, and Fe_3_O_4_, respectively; and the blue pigment is based on cobalt oxide (Co_3_O_4_) [9]. The particle density, the average diameter, and the particle shape of all pigments are presented on Table 1. The experimental program was defined to characterise the following properties: (i) water absorption by capillarity; (ii) accelerated carbonation; (iii) migration of chloride ions; (iv) resistivity; and (v) shrinkage. In addition, the colour change of the mortar when exposed to carbonation was measured through image processing.

Five reference mortars were designed: three white—WRM0.6, WRM0.5, and WRM0.4 with corresponding W/C ratios of 0.6, 0.5, and 0.4—and two grey—GRM0.6 and GRM0.5, with corresponding W/C ratios of 0.6 and 0.5. The reference mortars were designed by adopting the following proportions of binder powder materials to produce one cubic meter: 400 kg of CEM I 52.5R (white or grey) and 180 kg of limestone filler. The corresponding characterised densities, in kg/dm^3^, were 3.05 for white cement, 3.12 for grey cement, and 2.70 for limestone filler. No admixture was considered in WRM0.6 and GRM0.6 mortar, but the following proportions of ether–carboxylate superplasticizer were adopted, in percentage of cement weight, for the remaining mixtures, to assure consistency: 0.25% in mixtures with W/C = 0.5, and 0.75% in mixture with W/C = 0.4. The air content was considered 5% for mixtures with W/C of 0.6, and 4.5% for mixtures with W/C of 0.5 and 0.4. The remaining volume to produce one cubic meter was adjusted through sand (silicious sand 0/4 mm) proportion. The restoration mortars were formulated by adding black pigment at a rate of 2.5% and 4% of the cement weight to the white (WBP2.5% and WBP4%) and to the grey (GBP2.5% and GBP4%) reference mortar, WRM0.6 and GRM0.6, respectively. Pigment addition was considered by replacing the filler weight proportion. The same proportions (2.5% and 4% of cement weight) were also considered by adding yellow, red, and blue pigments (YEL2.5%, YEL4%, BLU2.5%, BLU4%, RED2.5%, and RED4%) to the reference white mortar WRM0.6. The formulation and characterisation of the produced mortar had been previously carried out, according to [4,5], with the mixture proportions of the reference mortar presented in Table 2, and the characterisation summarised in Table 3.

Specimens of the restoration mortar were prepared to be tested in laboratory, to evaluate their durability characteristics. Table 4 summarises the number and dimensions of specimens required for each test.

### 2.1. Durability Tests

#### 2.1.1. Capillary Water Absorption

Concrete structures are subject to water absorption by capillary phenomena mainly caused by rain. Water can transport aggressive agents and promotes conditions for steel reinforcement corrosion. Thus, low water absorption improves structure protection [10]. The capillarity water absorption test was performed following standard EN 1015-18 [11] and specification E-393 [12]. Water absorption by capillarity was studied by analysing the evolution of the water absorbed per unit surface of the specimens, as a function of the square root of time [13].

#### 2.1.2. Accelerated Carbonation

Carbonation results from the reaction of carbon dioxide (CO_2_) with concrete hydroxides, leading to a slow modification of structure and to a reduction in pH. Carbonation depth (Carb_D) occurs from the concrete surface and gradually advances inwards, forming a “carbonation front”, which separates two distinct zones in terms of pH: the non-carbonated zone, with a pH of circa 13, and the carbonated zone, generally with a pH below 9. The specimens were put in a chamber and subjected to an environment with high concentration of CO_2_, 5%, to accelerate carbonation, according to E391 [14]. The respective carbonation depth was measured, according to RILEM CPC-18 [15] recommendation. The carbonation front was determined by spraying an alcoholic solution of phenolphthalein. This indicator reveals the separation of the two zones with distinct pHs: carmine-coloured zones, with pH > 9 (non-carbonated), and non-coloured zone, with pH < 9 (carbonated).

Carbonation resistance, RC65 (kg·year/m^5^), for each mortar was determined using carbonation depth, C_di_, measured when exposed to an environment highly concentrated in carbon dioxide (Equation (1)), where C_acel_ is 90 × 10^−3^ kg/m^3^, t_i_ is the time exposed in years, and X_i_ has the same meaning as C_di_, i.e., carbonatation depth.

(1) $$R_{C65}=\frac{{2\;\times \;C}_{\mathrm{acel}\;}{\times \;t}_{i}}{X_{i}^{2}}$$

#### 2.1.3. Diffusion Coefficient Chlorides

Diffusion is the main process by which chloride ions progress through concrete, reach a considerable depth, and attack reinforcing steel bars NT build 492 [16,17]. The diffusion coefficient, D, is considered to be the most relevant parameter to characterise the resistance of concrete to the penetration of chloride ions [18]. The chloride migration test was performed according to E463 [19] specification, based on NT build 492 [17]. The diffusion coefficient of chlorides was determined based on Equation (2), where *D*_0_ is the diffusion coefficient in non-stationary regime, *U* is the absolute value of the potential difference (*V*), *T* is the average value of the initial temperature and end of anodic solution (°C), *L* is the thickness of the specimen (mm), *t* is the test duration (h), and *x_d_* is the average value of the depth of penetration (mm).

(2) $$D_{0}=\frac{0.0239\cdot \left(273+T\right)\cdot L}{\left(U-2\right)\cdot t}\cdot \frac{\left(x_{d}-0.0238\cdot \sqrt{\frac{\left(273+T\right)\cdot L\cdot x_{d}}{U-2}}\right)}{U-2}$$

#### 2.1.4. Electrical Resistivity

The electrical resistivity, ρ, of concrete is influenced by several factors, which are mostly interconnected. These factors can include temperature, moisture content, water/binder ratio, type of binder, type of aggregate, dimensions of test specimens, and influence of reinforcement, which are related to the durability parameters [20,21]. The result of this test is an important indicator for the development of corrosion in the reinforcement, in cases where the steel has easy access of oxygen. The electrical resistivity measurement was performed by the surface resistivity (SR) method according to the AASHTO T 358 [22] standard.

#### 2.1.5. Shrinkage

It is quite important to assure reduced shrinkage on the application of restoration mortar, to avoid cracks and to protect the reinforcement from the ingress of aggressive products that promote corrosion. The shrinkage test was carried out according to EN 1015-13 [23].

### 2.2. Chromatic Evaluation Due to Carbonation

Chromatic characterisation of the restoration mortar was performed through image processing using the HSV (from Hue, Saturation and Value) and CIELAB (International Commission on Illumination in L*a*b coordinates) colour spaces [5]. The analysis parameters selected in the HSV colour space were brightness (V) for the white and grey reference mortar, and saturation (S) for coloured mortar. In the CIELAB colour space, the parameters of luminosity (L*) and chromaticity (a* and b*) were selected to compute the colour differences (ΔE_00_) [24,25], between the end of the cure and the ages of specimen subject to accelerated carbonation.

### 2.3. Service Life

#### 2.3.1. Environmental Exposure XC (Carbonation-Induced Corrosion)

Laboratory tests to determine resistance to carbonation and to penetration of chlorides were used to evaluate the expected service life of the restoration mortar. These predictions were made based on the degradation and corrosion model used in the E465 specification [26]. The generally accepted model for the evolution over time of pre-stressed or reinforced concrete deterioration by corrosion of steel divides service lifetime into 2 periods: the initiation of corrosion, *t_ic_*, and the propagation of corrosion, *t_p_*. The minimum propagation period, *t_p_*, for each exposure class is defined in E465 [26] and the randomness of service life is considered to happen during *t_ic_*. The safety factor of service life, γ, is related to reliability classes adopted for each case, which is considered equal to γ = 2.3 for current structures. The values of *t_p_* (years) for each exposure class XC and *t_g_* of 50 years (RC2) are: >100 (XC1); 10 (XC2); 45 (XC3); 15 (XC4 (dry region)); 5 (XC4 (wet region)). The initiation time of corrosion, *t_ic_*, is computed by Equation (3).

*t_ic_* = γ.(*t_g_* − *t_p_*) (3)

where *t_g_* is the predicted service life.

The design period of initiation of structures repaired with the mortar can be determined though Equation (4) knowing X, which is equal to the cover defined, and the R_C65_ obtained for of each mortar.

(4) $$X=\sqrt{\frac{2\;\times \;0{.0007\;\times \;t}_{\mathrm{ic}}}{R_{C65}}}\;\times \;\sqrt{k_{0\;}{\times \;k}_{1}{\;\times \;k}_{2}}\;\times \;{\left(\frac{t_{0}}{t_{\mathrm{ic}}}\right)}^{n}$$

k_0_ is a factor related to the test conditions and is equal to 3, k_1_ is a factor related to relative humidity, k_2_ is related to concrete cure conditions and is 1 for a normalized cure, n is related to the influence of wetting/drying periods over time and t_0_ is the reference period, equal to 1 year.

#### 2.3.2. Environmental Exposure XS (Chloride-Induced Corrosion)

The propagation periods, *t_p_*, due to chlorides are specified in E465 [26], are different from carbonation, and assume for *t_g_* of 50 years (RC2) the following values (years): 0 (XS1 and XS3); 40 (XS2).

Based on the non-steady-state migration coefficients, D_0_, determined experimentally, the chloride diffusion coefficient, D, in m^2^/s, is determined for each developed mortar, using Equation (5).

D(t) = k D_0_.(t_0_/t)^n^ (5)

where k is a factor that takes into account the curing conditions, the relative humidity and the temperature, n is a factor that considers the chloride diffusion decrease during time, t_0_ is equal to 28 days, and t is the exposure time in days [26]. From the chloride diffusion coefficient, considering t equal to the design period of initiation, *t_ic_*, applying Equation (6) allows the determination of the minimum concrete cover required to provide the proper resistance against chloride-induced steel corrosion. Service life, *t_g_*, can also be predicted by Equation (3), but now X is equal to the cover defined, and the unknown variable is *t_ic_*.

(6) $$X=2.\xi .\sqrt{D.t_{ic}}$$

where ξ is a parameter related to the concentration of chlorides in the binder paste, and X is the considered cover.

## 3. Restoration Mortar Evaluation

### 3.1. Durability Tests

#### 3.1.1. Capillary Water Absorption Test

**Figure 1:**
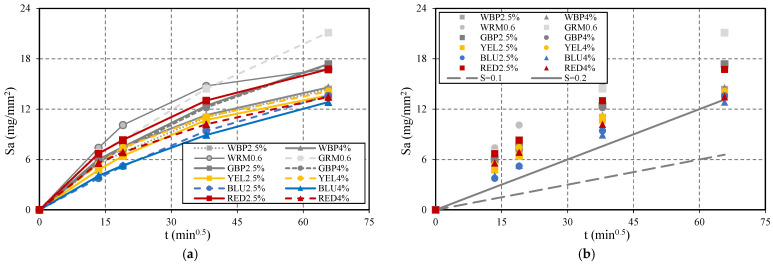
Capillary absorption coefficient Sa for the restoration mortar based on reference WRM0.6 and GRM0.6: (**a**) Capillary water absorption vs. square root of time; (**b**) fit of Sa values on the limits of quality for concrete.

The capillary water absorption was evaluated using the average of three 40 × 40 × 160 mm^3^ test specimens, registering the mass difference over time and the respective capillary water height at the specimen. Figure 1 shows the average capillary water absorption vs. the square root of time. The results show that the capillarity coefficients of all mortar with pigment incorporation are lower than the values of the reference mortars WRM0.6 and GRM0.6, during the entire test. In all mortars, water absorption by capillary occurs with greater intensity in the first 24 h, decreasing over time. The mortars with the addition of black pigment, WBP2.5% and WBP4%, have lower absorption values than reference WRM0.6, 25% at 24 h, and 15% at 72 h, respectively. In the case of adding black pigment to GRM0.6 (GBP2.5% and GBP4%), a decrease in absorption between 15% at 24 h and 18% at 72 h were registered. In particular, the blue mortar (BLU2.5% and BLU4%) are those with the lowest values of capillary absorption, 36% and 40% at 24 h, and 19% and 23% at 72 h. The mortar with yellow pigment incorporation, YEL2.5% and YEL4%, have an absorption decrease between 25% at 24 h and 17% at 72 h in relation to WRM0.6. Finally, the mortar with the addition of red pigment, RED2.5% and RED4%, present the capillary absorption lowest values, between 12% and 31% at 24 h and 0.25% and 25% at 72 h, in relation to white reference mortar WRM0.6. According to the classification of concrete quality due to absorption of water by capillarity [13], Sa (mg/mm^2^ × min^0.5^) above 0.2 is low quality, between 0.1 and 0.2 is medium quality, and below 0.1 is high quality. All mortar analysed (Figure 1b) is classified as low quality, but tends to improve with age.

The results show that the addition of black pigment causes a greater decrease in the reference mortar produced with white cement in relation to that produced with grey cement. In particular, mortar with the addition of blue pigment has the lowest capillary absorption value. In all cases, the first 24 h are the most relevant for water absorption by capillary. The capillary absorption coefficient decreases with the addition of pigments, as they also contribute to filling gaps in the mortar, resulting in a denser mortar and, consequently, less water absorption. The pigment in mortar causes a microfiller effect due to the size and shape of the pigment particle, which is finer and has a larger specific surface area than cement.

#### 3.1.2. Accelerated Carbonation Test

**Figure 2:**
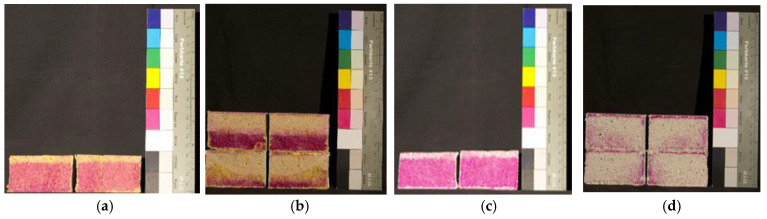
Carbonation depth: (**a**) YEL4% 7 days; (**b**) YEL4% 128 days; (**c**) WRM0.6 7 days; (**d**) WRM0.6 128 days.

The carbonation depth was measured at regular intervals of 7, 14, 28, 56, and 128 days of exposure (Figure 2). Mortar WRM0.5, WRM0.4, and GRM0.5 have the smallest carbonation depth, being about 11, 22, and 4 times smaller than that of WRM0.6 and GRM0.6, respectively. WRM0.6 at 128 days is seriously carbonated and the evolution follows a linear trend (Figure 2d). In the case of GRM0.5, the values tend to slightly increase, showing that reduced W/C ratio significantly affects the propagation of carbon dioxide into concrete.

**Figure 3:**
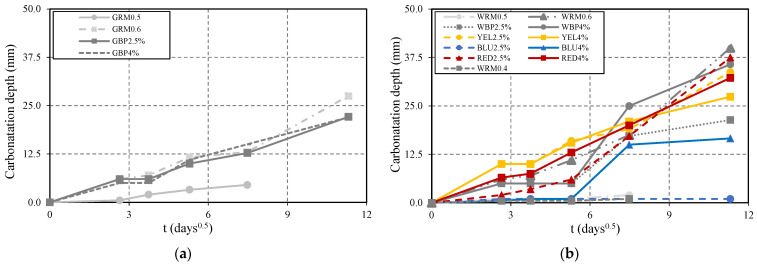
Evolution of the carbonation depth with the square root of time t (days): (**a**) GRM0.6, GRM0.5, GBP2.5%, GBP4%; (**b**) WRM0.6, WRM0.5, WRM0.4, WBP2.5%, WBP4%, YEL2.5%, YEL4%, BLU2.5%, BLU4%, RED2.5%, RED4%.

Mortar with the addition of black pigment (WBP2.5% and WBP4%) has a carbonation depth similar to WRM0.6, between 7 and 28 days. In the case of GBP, carbonation depth of up to 56 days is identical to WRM0.6 (Figure 3a). Both tend to have shallower carbonation depths at older ages. YEL mortar has the highest carbonation depth, being approximately 2 times higher than that of WRM0.6 at 28 days, but tends to have similar carbonation depth at longer ages. The cobalt oxide pigment was revealed to have a large influence in reducing carbonation, since BLU has the lowest values, with no apparent changes in BLU2.5% between 7 and 128 days. However, in BLU4%, a very significant increase occurs after 56 days, and there are non-major changes at 128 days. This does not provide an evident trend, therefore a repetition of this test with several pigment proportions will be conducted in the near future to clarify this issue. The difference between 2.5% and 4% of red pigment is noticeable in RED mortar. The RED2.5% has lower carbonation values up to 28 days in relation to the WRM0.6, while in the RED4%, the values are similar (Figure 3b). The carbonation coefficient, which reflects the evolution of the carbonation depth, is determined from the slope obtained by adjusting a line to the points obtained from Carb_D vs. square root of time [15], as in Figure 3.

#### 3.1.3. Diffusion Coefficient Chlorides

**Figure 4:**
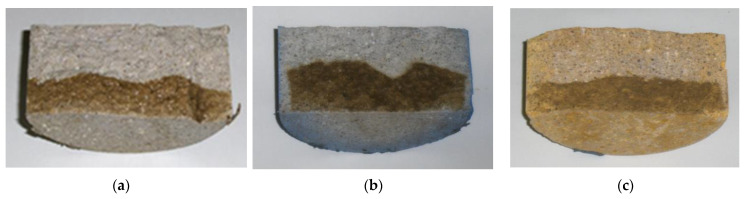
Average value of the depth of penetration xd according to AgNO_3_ sprinkling on samples: (**a**) WRM 56 days; (**b**) BLU4% 28 days; (**c**) YEL2.5% 56 days.

Cylindrical samples were prepared (Table 3) and afterwards subjected to ionic migration. The depth of chloride diffusion into mortar was measured by the colorimetric method, after testing and cutting the sample and spraying each side with silver nitrate solution; the colour changes to silver in the presence of chloride ions (Figure 4).

**Figure 5:**
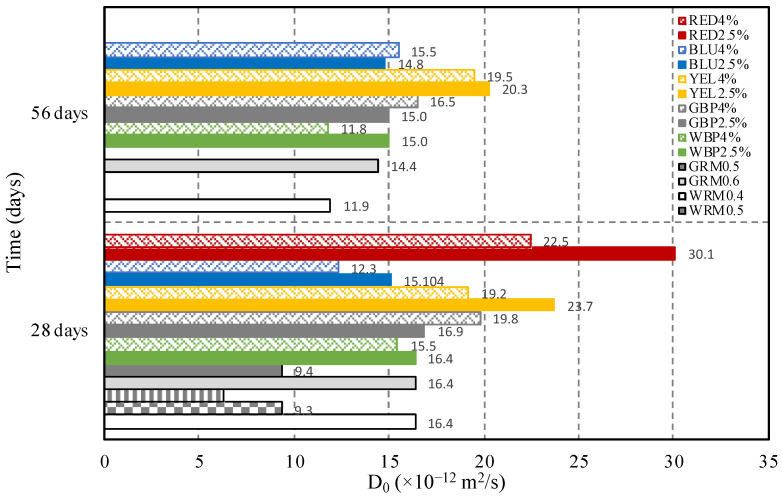
Diffusion coefficients of chlorides (D_0_), non-stationary migration tests (28 and 56 days).

The results of the chloride diffusion coefficient in the non-steady state (D_0_) are shown in Figure 5. D_0_ values were assessed at 28 and 56 days for the restoration mortars WRM0.6, GRM0.6, WBP2.5%, WBP4%, GBP2.5%, GBP4%, YEL2.5%, YEL4%, BLU2.5%, and BLU4%; and only at 28 days for WRM0.5, WRM0.4, GRM0.5, RED2.5%, and RED4%. The coefficients were compared at the same age (28 days), and the values of both reference mortars with W/C ratio of 0.5 decrease by 43% in relation to the reference mortar with W/C of 0.6. In the case of WRM0.4 the decrease is higher, with a reduction of 62% in relation to WRM0.6, due to the lower W/C ratio. The W/C ratio influences the porous structure of the mortar and, consequently, affects the penetration of chloride ions into the matrices. The chloride diffusion coefficient decreases when the W/C decreases, regardless of whether the reference mortar is produced with white or grey cement.

The addition of 2.5% black pigment (WBP2.5%) to the WRM0.6 does not cause any change, presenting values equal to the WRM0.6 at 28 days. However, at 56 days, an increase of 26% was registered. The addition of 4% (WBP4%) shows a slight decrease of 5% at 28 days and 1.0% at 56 days. In the case of the addition of black pigment to GRM0.6, the GBP2.5% mortar presents identical values, increasing by 3% at 28 and 56 days; GBP4% presents values higher than those of the GRM0.6 mortar, 21% at 28 days, and 14% at 56 days.

The incorporation of the yellow pigment, 2.5% and 4%, in the WRM0.6, results in mortar with higher permeability for chloride migration. Thus, it reduces the resistance to chloride ion migration by 45% and 17% at 28 days, increasing the reduction at 56 days to values of 70% and 64%, respectively. The increase of D_0_ coefficient in mortar with the yellow pigment is probably related to the characteristics of the pigment particles (Table 1). The particles shape can influence the reduction of the packing density regarding the internal arrangement of the smaller particles. The obtained values for blue mortar with 2.5% and 4% of pigment present lower values than the reference WRM0.6 mortar—8% and 24% lower, respectively. It seems that the cobalt oxide pigment, which is composed of smaller particles (Table 1), promotes the formation of a denser mortar matrix that leads to a higher resistance to chloride diffusion.

The mortar with the addition of 2.5% red pigment has the highest chloride migration, increasing diffusion by 84% at 28 days, with an increase of approximately 37% for the addition of 4% of pigment at the same age. However, resistance to chloride changes over time and the benefits compared to reference mortar are not always the same over time. For example, at 56 days, improvements were registered for WRM0.6, GRM0.6, and GBP4%, since D_0_ reduces by about 13 to 27% in comparison to at 28 days.

#### 3.1.4. Electrical Resistivity

**Figure 6:**
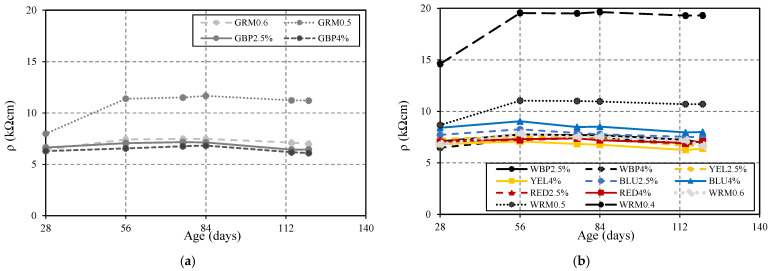
Electrical resistivity as a function of age: (**a**) GRM0.6, GRM0.5, GBP2.5%, GBP4%; (**b**) WRM0.6, WRM0.5, WRM0.4, WBP2.5%, WBP4%, YEL2.5%, YEL4%, BLU2.5%, BLU4%, RED2.5%, RED4%.

The test was carried out according to the AASHTO T 358 [22] standard on cylindrical test pieces with a diameter of 100 mm and a length of 200 mm. The equipment used was Proceq’s Resipod, a 4-point Wenner probe designed to measure electrical resistivity. Mortar resistivity was assessed at 28, 56, 76, 84, and 120 days of age (Figure 6).

From 28 to 56 days, all mixtures tend to increase ρ values, with a notable difference between GRM0.6 and GRM0.5 (Figure 6a) and between WRM0.6, WRM0.5, and WRM0.4, (Figure 6b) due to the difference in W/C. However, the difference is less pronounced in mixtures with the addition of red and black pigments. After 56 days, no significant evolution was recorded, since there are no pozzolanic additions, so there is no reactivity at older ages. For that reason, there is no refinement of the porous structure, and the values tend to remain constant. It is quite evident that W/C reduction from 0.6 to 0.5 increases the resistivity by about 40% at 56 days, for WRM0.5 and GRM0.5. In the case of WRM0.4, where the W/C reduces from 0.6 to 0.4, the increase in resistivity is of approximately 160%. The pigments used have the opposite effects, since yellow and red pigments tend to slightly reduce resistivity, whereas blue pigment tends to increase it by approximately 16% (Figure 6b). The length/width ratio of the iron oxide particles with the needle shape is probably the main reason for the decrease in resistivity. It is noticeable that the influence of those parameters in increasing resistivity is similar to the effects registered in the reduction of chloride diffusion coefficient.

#### 3.1.5. Shrinkage

**Figure 7:**
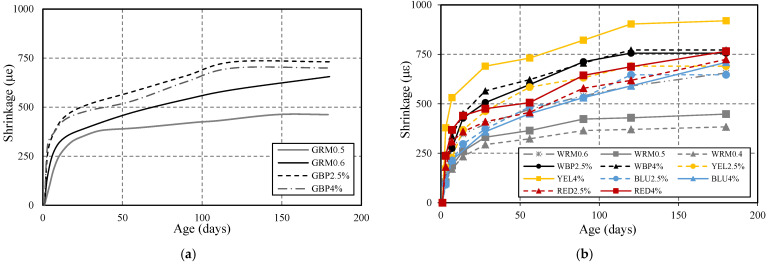
Shrinkage evolution of the produced mortar, with: (**a**) GRM0.5, GRM0.6, GBP2.5%, GBP4%; (**b**) WRM0.6, WRM0.5, WRM0.4, WBP2.5%, WBP4%, YEL2.5%, YEL4%, BLU2.5%, BLU4%, RED2.5%, RED4%.

Shrinkage test was carried out on prismatic specimens (Table 4), measured with a proper analogic transducer between the ends of the specimens, using stainless steel inserts. Mortar shrinkage was evaluated at 1, 3, 7, 14, 28, 56, 90, 120, and 180 days of age. The obtained values correspond to the average results of 3 specimens and are plotted in Figure 7. The evolution is similar for all mixtures, with a pronounced increase at early stages and a moderate increase after 14 days. WRM0.5 and WRM0.4 promoted an important reduction in shrinkage, by about 22% and 33%, respectively, when compared to WRM0.6. In the case of GRM0.5 (Figure 7a) the reduction is 22% in relation to GRM0.6, presenting similar values to WRM0.5. The incorporation of black pigment, regardless of the percentage (2.5% or 4%), shows the same increase of approximately 31% compared to WRM0.6. In GBP, the variation in the percentage of black pigment (2.5% or 4%) is different, showing increases of 22% and 49%, respectively, in relation to GRM0.6. When red pigments RED2.5% are incorporated, the increase is less pronounced, with a value of 7%, increasing to 19% if addition of pigment is higher than RED4%, in relation to WRM0.6. The incorporation of a yellow pigment with 4% content shows an effective increase in shrinkage, 67% greater than reference WRM0.6, at 90 days. By contrast, the blue pigment promotes shrinkage reduction compared to WRM0.6, with values of 2% and 9%, respectively, for BLU2.5% and BLU4%. The evolution of shrinkage curves is similar; however, the amplitude is mainly affected by the W/C ratio and by the pigment type, where black and yellow pigments tend to increase shrinkage and blue pigment tends to reduce shrinkage. The main reason for this influence is probably the interference of pigment shape and dimensions on the microstructure and on the packing density of the mixtures. This issue must be further investigated in the future, together with a possible chemical reaction.

### 3.2. Chromatic Evaluation Due to Carbonation

**Figure 8:**
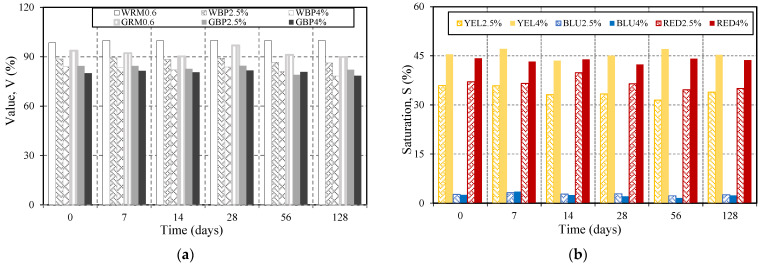
Chromatic characterisation: (**a**): Value (WRM0.6, WBP2.5%, WBP4%, GRM0.6, GBP2.5, GBP4%); (**b**): Saturation (YEL2.5%; YEL4%; BLU2.5%; BLU4%, RED2.5%, RED4%).

**Figure 9:**
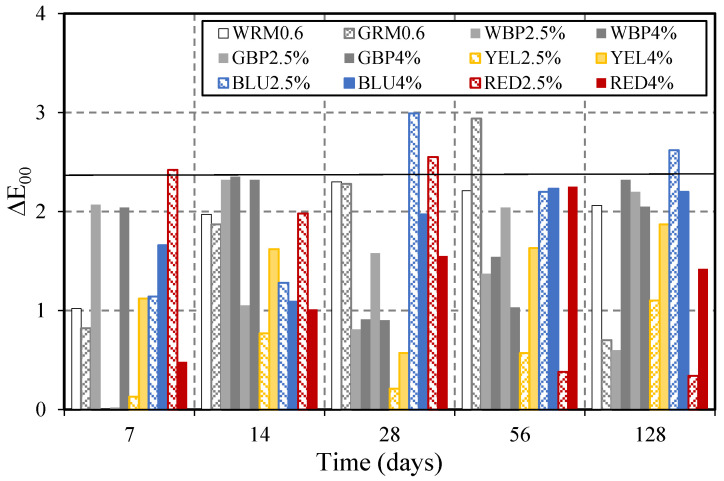
ΔE_00_ variations of mortar series produced with cements.

Chromatic characterisation of the restoration mortar was performed through image processing using the HSV and CIELAB colour spaces [1,2]. The analysis parameters selected in the HSV colour space were brightness (V) for white and grey mortar, and saturation (S) for coloured mortar (yellow, red, and blue). In the CIELAB colour space, the parameters of luminosity (L*) and chromaticity (a* and b*) were selected to compute the colour differences (ΔE_00_) [24], between the end of the cure and the stages where the specimen were subject to accelerated carbonation. The brightness parameter (V) in the white reference mortar WRM0.6 remained unchanged (98%) for all ages (Figure 8a). The saturation parameter (S) in coloured mortar after 7, 4, 28, 56, and 128 days of accelerated carbonation were shown in Figure 8b. Additionally for those cases, the colour remains stable over time. With the aim of detecting small colour differences, ΔE_00_ was computed and plotted in Figure 9. For values bellow 2.3, defined as JND (Just Noticeable Difference) [24], it is accepted that the human eye is not able to perceive colourimetric differences. The values show a variation of ΔE_00_ below the JND, except for BLU2.5% at 28 and 128 days, RED2.5% at 28 days, and GRM0.6 at 56 days. This change may have been caused by the release agent. In general, the results show that carbonation did not cause colour changes in any mortar that was perceptible to the human eye.

### 3.3. Expected Service Life

#### 3.3.1. Environmental Exposure XC (Corrosion Induced by Carbonation)

Table 5 presents the mean values of the carbonation resistance, R_C65_, considering only the results at 56 and 128 days, because at these stages the error related to the measurement of the carbonation depth is lower. At early stage, the C_di_ has a small value, and a small difference has a huge impact on R_C65_.

**Figure 10:**
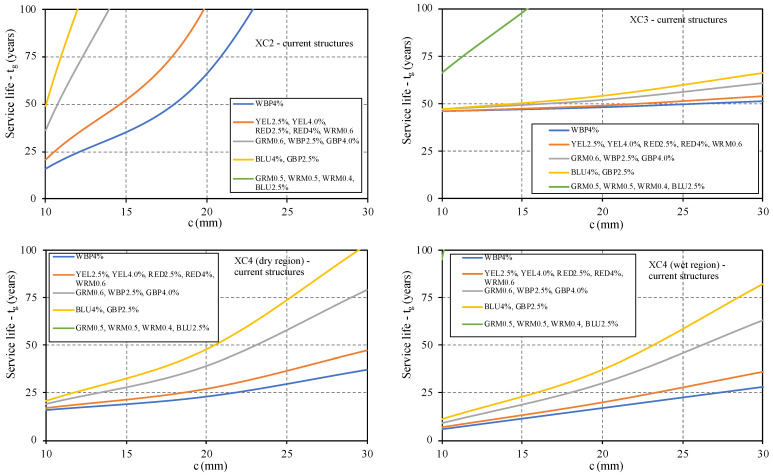
Prediction of the service life for current structures, classes XC.

The service life was computed for different exposure classes (for current structures), for a cover equal to 10, 20, and 30 mm (see Figure 10). The results are only conditioned by the requirements to protect the steel reinforcement against corrosion, not taking into account the fire resistance and the proper transmission of bond stresses between the rebars and the surrounding mortar.

For the environmental conditions related to exposure classes XC2 and XC3, a 20 mm cover using any of the developed mortar is enough to ensure a service life of at least 50 years. This means that the carbonation resistance, R_C65_, of the studied mortar is suitable for these conditions. For exposure classes XC4, corresponding to structures exposed to high air humidity or those that are in contact with water, it is necessary to increase the cover to more than 20 mm to ensure the proper steel protection, and for several mortars, such as WBP4%, YEL2.5%, WRM0.6, 30 mm of cover is not enough to ensure 50 years of service life. However, even in those cases, with lower carbonation resistance, R_C65_, it is demonstrated that 25 can be achieved using covers with a thickness between 20 and 30 mm. The mortars GRM0.5, WRM0.5, WRM0.6, and BLU2.5% have huge carbonation resistance, R_C65_, meaning that they have excellent durability performance in these environments and are suitable to be used as restoration mortar. However, the results need to be corroborated with more tests.

The results also show that the percentage of pigment used does not significantly affect carbonation resistance, R_C65,_ and consequently the service life, although small variations were recorded when the quantity of pigment is changed. Therefore, for practical proposes, it can be stated that small changes to mortar composition in terms of pigment percentage can be made without compromising the required protection. On the other hand, the type of used pigment already has an influence that cannot be neglected; for example, the BLU4% mortar has a carbonation resistance, R_C65,_ three times higher than the WBP4% mortar.

#### 3.3.2. Environmental Exposure XS (Chloride-Induced Corrosion)

The non-steady-state migration coefficients, D_0_, experimentally determined, are presented in Table 6.

**Figure 11:**
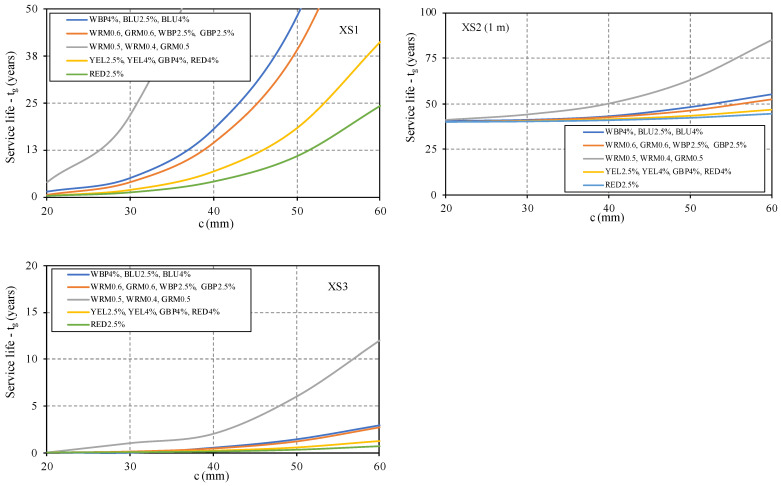
Prediction of the service life for current structures, classes XS.

The prediction of the expected service life considering the corrosion of the steel reinforcement induced by chlorides, depending on the considered cover, was also performed by groups of mortar with similar chloride results (Table 7 and Figure 11), based on the worst value of chloride diffusion coefficient in each group.

The results show that the mortar does not provide proper corrosion resistance of the steel reinforcement in environments with chlorides. Even in exposure classes XS1, the minimum cover required to ensure an adequate protection is too high to be applied in practical situations. Therefore, in these environmental conditions, with structures exposed to air sea salts, it is necessary to provide additional steel protection to increase the restoration durability. Classes XS3, tidal splash, and spray zones correspond to a very aggressive environment, but the probability of needing to repair cementitious mortar with high aesthetic requirements is low. The zones exposed to XS2 have higher service life due to the propagation of corrosion periods *t_p_*, and for underwater zones this period is high. The corrosion of steel and degradation of concrete only occurs during the propagation period. The initiation period *t_ic_* corresponds to the time necessary for carbon dioxide or chlorides to penetrate the mortar cover, through the open pores system, and reach the steel. In this study, as already mentioned, the minimum values of *t_p_*, according E465 [26], were adopted and were constant for each exposure class. This means that the type and quality of mortar or concrete affects only the initiation period *t_ic_*, and consequently the extent of service life. The developed mortar clearly provides initiation periods of close to zero, meaning that chlorides penetrate the mortar cover very easily.

## 4. Conclusions

An experimental study to evaluate the durability characteristics of restoration mortar, produced through the addition of pigments to reference cement-based mortar, was conducted. This includes the analysis of mortar colour evolution when exposed to carbonation, through image processing. Finally, the relationship between cover depth and service life was estimated and analysed. The main conclusions in each of these three topics are the following:

(a) Indicators of durability performance

The incorporation of pigments in both white and grey cement mortar improves performance by reducing water absorption. The addition of pigment results in a denser mortar and, consequently, the capillary absorption coefficient decreases. In all mortar, capillary action occurs with greater intensity in the first 24 h, and decreases over time.

The carbonation depth changes significantly for mortar with different cement–water (W/C) ratios and with the addition of pigment. The worst performance concerning carbonation was the reference mortar with W/C of 0.6, i.e., those that had higher carbonation depths, as expected. Those values were insignificant for reference mortar with W/C ratios of 0.5 and 0.4, confirming that the W/C ratio decrease significantly reduces the entry of carbon dioxide into concrete, because porosity and permeability is reduced. The cobalt oxide pigment (blue) appears to have a high influence on carbonation reduction. However, results do not provide a clear trend, and more tests with several pigment proportions will be conducted in the near future to clarify this issue.

The chloride diffusion coefficient is also influenced by the W/C ratio and the type and percentages of added pigment. This coefficient decreases when the W/C decreases, regardless of the reference mortar, due to the alteration of the porous structure of the mortar matrices. Regarding the addiction of pigment: (i) black pigment has no influence; and (ii) yellow pigment reduces the chloride ion migration resistance depending on the percentage. These results are probably due to the different shape (acicular) and size of the yellow pigment particles, which influence the internal arrangement of the mortar, increasing the packing density; (iii) blue pigment also reduces the chloride ion migration resistance. It seems that the cobalt oxide pigment, composed of smaller particles, promotes the formation of a denser mortar matrix that leads to a higher resistance to chloride diffusion. The tests to measure the electrical resistivity of mortar corroborate this trend.

(b) Colour change and shrinkage

The colour variations of the restoration mortar, when exposed to accelerated carbonation with different test times, are minor and generally not perceptible to the human eye.

The evolution of shrinkage curves is similar in all mortar; however, the amplitude is mainly affected by W/C ratio and by pigment type, where black and yellow pigment tends to increase shrinkage and blue pigment tends to reduce shrinkage.

(c) Service life

All restoration mortar has adequate carbonation resistance for a service life of 50 years, for all exposure XC classes. It must be highlighted that some mortar, namely blue mortar (BLU), has excellent performance regarding this issue, probably due to the shape and size of pigments. For example, the blue mortar BLU4% has a resistance to carbonation approximately three times higher than the yellow mortar YEL4%. Furthermore, small variations of the percentages of pigments do not significantly affect the resistance to carbonation and, for practical purposes, it can be stated that those minor changes of mortar composition do not compromise the intended protection.

Mortar produced with a W/C ratio of 0.6 does not provide an adequate protection to chloride-induced steel corrosion. Even for the XS1 exposure class, the minimum cover required is too high to be applied in current situations. The reduction of W/C ratio to 0.5 increases the resistance to chloride diffusion and, consequently, increases the service life expected. For XS exposure classes, it is recommended to predict and schedule regular maintenance interventions, depending on the adopted cover.

## Figures and Tables

**Table 1:** Characteristics of yellow, red, black, and blue pigments.

Pigment	Phases	Density (kg/dm^3^)	Average Particle Diameter (nm)	Particle Shape
Yellow	FeHO_2_	4.25	48.73	acicular
Red	Fe_2_O_3_	5.25	69.22	spherical
Black	Fe_3_O_4_	5.18	67.02	spherical
Blue	Co_3_O_4_	6.44	46.53	prismatic

**Table 2:** Mix Proportions of the restoration reference mortar.

Mortar		Cement (kg/m^3^)	Filler (kg/m^3^)	Sand (kg/m^3^)	Water (kg/m^3^)	Superplast. (kg/m^3^)	W/C
Colour	Reference						
	WRM0.6	400	180	1347	240	0.00	0.6
White	WRM0.5	400	180	1463	200	1.00	0.5
	WRM0.4	400	180	1564	160	2.80	0.4
Grey	GRM0.6	400	180	1355	240	0.00	0.6
	GRM0.5	400	180	1471	200	1.00	0.5

**Table 3:** Characterisation properties of reference mortar.

Mortar		Spread Diameter (cm)	Air Content (%)	Strength (MPa), at 28 Days	
Colour	Reference			Flexural	Compressive
	WRM0.6	11.5	5.0	7.8	59.0
White	WRM0.5	11.0	4.6	8.3	73.0
	WRM0.4	10.5	4.3	9.8	87.2
Grey	GRM0.6	12.0	4.8	7.7	51.6
	GRM0.5	11.5	4.5	7.3	66.8

**Table 4:** Summary of the tests performed to study the durability of the restoration mortar.

Tests	Samples	Cure	Age
Capillary water absorption	40 × 40 × 160 mm 3 samples	Water curing (20 °C ± 2 °C)	Test at 3, 6, 24, and 72 h
Accelerated carbonation test	40 × 40 × 160 mm 3 samples per age	14 days in water curing (T = 20 °C); 14 days in air dry (HR = 50% and T = 20 °C)	Test at 7, 14, 26, 56, and 128 days
Non-stationary migration test	Ø 100 × 50 mm 2 samples per age	7 days of water curing (T = 20 °C); Cut; 21 days of air dry (HR = 50% and T = 20 °C)	Test at 28 and 56 days
Shrinkage	40 × 40 × 160 mm 2 samples	Air dry curing (HR = 50% and T = 20 °C)	Measure at 1, 3, 7, 14, 28, 56, 90, 120, 180 days
Resistivity	Ø 100 × 200 mm 1 sample	Water curing (20 °C ± 2 °C)	Measure at 28, 56, 76, 84, 114, and 128 days

**Table 5:** Values of carbonation resistance for each mortar, RC65 (kg·year/m^5^).

WRM0.6	WRM0.5	WRM0.4	GRM0.6	GRM0.5	WBP 2.5%	WBP 4%	GBP 2.5%	GBP 4%	YEL 2.5%	YEL 4%	BLU 2.5%	BLU 4%	RED 2.5%	RED 4%
65	>1000	>1000	123	>1000	115	47	149	127	66	73	>1000	176	69	65

**Table 6:** Values of the diffusion coefficient in non-stationary regime, D_0_ × 10^−12^ (m^2^/s).

WRM0.6	WRM0.5	WRM0.4	GRM0.6	GRM0.5	WBP 2.5%	WBP 4%	GBP 2.5%	GBP 4%	YEL 2.5%	YEL 4%	BLU 2.5%	BLU 4%	RED 2.5%	RED 4%
16.4	9.4	6.2	16.4	9.4	16.4	15.4	16.9	19.8	23.7	19.2	15.1	12.3	30.1	22.5

**Table 7:** Prediction of service life, t_g_, exposure classes XS ^1^.

Group of Mortar	Cover (mm)	Current Structures			
		XS1 ^2^	XS2 ^3^		XS3 ^4^
			1 m	1.4–25 m	
{WBP4%, BLU2.5%, BLU_4%}	20	1	40	40	0
	30	5	41	41	0
	40	18	43	42	1
	50	48	48	46	1
	60	>100	55	51	3
{WRM0.6, GRM0.6, WBP2.5% GBP2.5%}	20	1	40	40	0
	30	4	41	41	0
	40	14	43	42	0
	50	39	46	45	1
	60	88	52	49	3
{WRM0.5, WRM0.4, GRM0.5}	20	4	41	41	0
	30	22	44	43	1
	40	79	50	47	2
	50	>100	63	56	6
	60	>100	85	71	12
{YEL2.5%, YEL4%, GBP4%, RED4%}	20	0	40	40	0
	30	2	41	40	0
	40	7	42	41	0
	50	18	43	43	1
	60	41	47	45	1
	20	0	40	40	0
	30	1	40	40	0
{RED2.5%}	40	4	41	41	0
	50	11	42	42	0
	60	24	44	43	1

^1^ In this analysis, the worst-case scenario was considered, i.e., the structures are located on the coast. ^2^ Structures exposed to air sea salts. ^3^ Permanently submerged structures. ^4^ Structures in tidal splash and spray zones.

## Data Availability

Data sharing not applicable.
